# The Plague of Provence (1720–2) and debates in Britain on the cross-species transmission of disease

**DOI:** 10.1017/mdh.2025.10013

**Published:** 2025-07

**Authors:** Neil Murphy

**Affiliations:** Northumbria University, Newcastle, UK

**Keywords:** Cattle, Plague, Animals, Britain, Provence, France, quarantine, contagion, murrain, rinderpest

## Abstract

While the plague of Provence is the most studied outbreak of the disease in early modern Europe, there is little in the extensive historiography on this topic about fears of the cross-species transmission of disease which re-emerged in the early eighteenth century because of events in southwestern France. Concerns about the interplay between cattle murrains and human plague resurfaced in the early eighteenth century because the plague of Provence followed an outbreak of cattle disease which swept across Europe and killed tens of thousands of animals. This article focuses on the debate about the spread of contagious diseases between species which occurred in Britain during this time. Links between the health of animals and that of humans became objects of heated discussion especially following the issuing of the 1721 Quarantine Act, which was designed to prevent the plague currently ravaging southwestern France from taking hold in Britain. It then considers the different beliefs regarding contagion and the transmission of diseases between different species during the plague of Provence. While focusing on the richly documented and highly revealing discussions in early eighteenth-century Britain about the interplay between plague in cattle and plague in humans, it also utilises materials from earlier centuries to examine more fully how early modern populations understood the relationship between plague in humans and cattle murrains.

In 1720, a ferocious outbreak of plague hit Provence. By the time it had abated, the disease had killed 126,000 people. As one of the last major outbreaks of plague in Western Europe, the Plague of Provence has long attracted the attention of historians.^1^ It is the most studied outbreak of plague in early modern Europe and has been the subject of a dozen or more books and numerous articles.^2^ Despite the high volume of publications on the Plague of Provence, these works say little regarding the concerns about the potential for cross-species transmission of disease, which re-emerged in the early eighteenth century as a result of this outbreak. Indeed, there is little work on fear about the spread of disease from animal to human in the extensive historiography on plague (and of contagious diseases more widely) in late medieval and early modern Europe. Although some historians of plague have recently started to consider the impact which outbreaks had on animal populations, these comments are confined to urban disease-control measures, and they do not seek to examine past societies’ concerns about links between human and animal diseases.^3^ If epizootics are mentioned at all in the large literature on plague, it is largely in terms of the fleas jumping from dying rats to spread the disease to humans.^4^ This connection between plague in rats and plague in humans was observed in China in the 1890s during the third pandemic of plague and then retrospectively applied to the second pandemic (fourteenth to nineteenth centuries).^5^ However, connections between rat epizootics and the outbreak of plague amongst humans were not apparent to late medieval and early modern populations. Rather, as we shall see, people of those times were particularly concerned about the potential for the transmission of disease between humans and domesticated animals, especially cattle. Furthermore, while there is a growing body of literature on pre-modern animal diseases, these works principally focus on the continent-wide cattle plagues which struck Europe in the early fourteenth century^6^ and then again in the later eighteenth and nineteenth centuries.^7^ These studies principally focus on the cattle (and the humans who tended to them) and the wider impact of the outbreaks, especially in economic terms, rather than consider how contemporaries conceived of connections between animal and human diseases.^8^ While animal murrains and outbreaks of plague in humans are largely studied separately, this article demonstrates that past societies considered them together, and often as manifestations of the same disease. It argues that fears about the cross-species transmission of disease arose not simply because pre-modern populations noted that both humans and animals alike suffered from contagious diseases but because outbreaks of disease in different species often occurred concurrently.

This article focuses on the debate about the spread of contagious diseases between species, which occurred in Britain during the Plague of Provence. Concerns about the interplay between cattle murrains and human plague resurfaced in the early eighteenth century precisely because of the apparent co-occurrence of the two diseases. Between 1709 and 1720, an outbreak of cattle disease (probably rinderpest) swept across Europe and killed tens of thousands of animals; this was followed by the irruption of a devastating outbreak of plague amongst human populations in Provence in the early 1720s. Although Britain lay distant from Provence, George I’s government was concerned that the plague could spread to its shores. Links between the health of animals and that of humans became objects of heated discussion, especially following the issuing of the 1721 Quarantine Act, which was designed to prevent the plague currently ravaging southwestern France from taking hold in Britain. Although a range of studies examine the contagionist debates of the 1720s, they say little or nothing about the prominent position that ideas about the transmission of disease between different species occupied in these discussions.^9^ Yet the leading advocates on both sides of the debate in the early 1720s deployed opposing views on the cross-species transmission of disease to support their claims, such as Richard Mead, whom the government appointed to implement quarantine regulations based around ideas of contagion to prevent the Plague of Provence from reaching Britain, and Richard Blackmore who opposed such measures. Although historians have examined their writings, they have largely omitted from their analyses of these debates the extensive discussion of animal diseases these authors put forward in these works. This is a significant omission as these views about animals formed an important element of their respective claims regarding ideas about contagion.

While focusing on the richly documented and highly revealing discussions in early eighteenth-century Britain about the interplay between plague in cattle and plague in humans, this article also utilises materials from earlier centuries, especially from the sixteenth and seventeenth centuries – both from Britain and continental Europe – to examine more fully how early modern populations understood the relationship between plague in humans and cattle murrains.^10^ Indeed, those people writing about the Plague of Provence in the 1720s looked to past outbreaks of plague in cattle and plague in humans and discussed them at length in their works. This article particularly utilises plague tracts, a form of medical treatise which emerged during the Black Death in the mid-fourteenth century and continued to be used until the nineteenth century.^11^ Beyond these sources, it employs a variety of other medical and scientific texts, from manuals of husbandry to direct observations of murrains, as well as sermons and other religious texts. These help reveal wider understandings of the origins of cattle plague, especially those derived from biblical content which sat beside – and intersected with – various medical understandings of disease. From the sixteenth century especially, medical knowledge about the origins of disease and its effects on human and animal bodies was changing, with older theories about the role of miasmas, ultimately derived from the ancients and especially Galen, being challenged by – or adapting to encompass – ideas regarding contagion both between members of the same species and between different species.

The article begins by outlining the central role which cattle occupied in society before moving on to consider how pre-modern European populations conceived of co-occurrences of contagious disease in humans and animals. Building on this contextual foundation, the article proceeds to examine the specific debates which occurred around the 1721 Quarantine Act in Britain and demonstrates that discussions on the aetiology of cattle plague occupied a prominent part in these debates, an element which has been overlooked in the historiography of this subject. It then considers the different beliefs regarding ideas about contagion and the transmission of diseases between different species during the Plague of Provence. The final part of the article demonstrates that views about cattle as the vectors of disease in humans voiced in response to the Plague of Provence sat alongside nascent understandings of how humans could transmit diseases to cattle and other animals in what would be termed today as reverse zoonosis.

## Cattle in pre-industrial Europe

Diseases of domesticated animals – and cattle in particular – were of great concern to the populations of pre-industrial Europe. As Keith Thomas observed, cattle, along with horses, underpinned pre-modern European civilisation.^12^ Before the development of modern mechanised agriculture in the nineteenth century, cattle were used to work the fields while their manure fertilised the land. The onset of the second pandemic of plague in the fourteenth century led to a rise in cattle populations in Europe as their labour became more necessary than ever, given the high mortality rate of the Black Death and its subsequent waves.^13^ Beyond their role in arable farming, cattle provided sustenance in the form of dairy produce and, less regularly (at least for the poorer classes), through their meat. They were often the most valuable possessions in the households of rural working families. In medieval and early modern Europe, people lived in much closer proximity to cattle than they do now. They shared the same spaces and laboured together in the fields. Cattle were held in high particular esteem, and their loss was seen as an especially bad disaster. In the *Godly ballad of the just man Job* (1658), the death of cattle is likened to the death of children as one of the greatest disasters which could befall a person.^14^ Cattle underpinned the wider functioning of society so much that in the comprehensive anti-plague measures devised by Elizabeth I’s privy council in 1578 (and re-issued by subsequent monarchs), a rare exception was made for people to leave infected houses ‘for the seruing of their cattel, and manuring of their ground’.^15^ Wishing that the plague would take a person or their cattle was a common curse in the seventeenth century, with the independent minister Matthew Mead noting in his 1665 work, *Solomon’s prescription for the removal of the pestilence*, ‘how many times have People in their Execrations wish’t, That the Pox and the Plague might take Themselves, their Children, Servants, or Cattle.^16^’ For George Wither, the English puritan poet, a healthy kingdom was one free from plague and crop blight and where ‘No Rots or Murraines have our Cattell kild…[and] Our breeding Cattell fill both street and way’.^17^

Lise Wilkinson, in her study of rinderpest outbreaks in the eighteenth century, states that before the establishment of the first veterinary school in the 1760s, ‘the care of cattle had been the exclusive province of unenlightened shepherds and cow-leeches’. ‘Veterinary medicine’, she continues, ‘was non-existent both in theory and practice apart from a few treatises on the horse, reflecting the preoccupation of the reading classes with this important transport animal’.^18^ Yet significant attention was paid to cattle illnesses in earlier centuries, not least because the health of these animals was essential for the functioning of society, and, as we shall see, many of the developments Wilkinson sees as manifestations of the eighteenth century had earlier roots, including ideas about quarantine and infection amongst cattle. In his 1534 *Book of Husbandry*, which Lucinda Cole notes was ‘the first text [in England] to deal with mass animal die-offs’, Anthony Fitzherbert advised that during a cattle plague, infected animals were to be buried deep so ‘that noo dogges may come to the caryen. For as many beastes as feleth the smelle of that caryen, are lykely to be enfecte’, to which end he also said that the disease could be spread through infected hides.^19^ Tanning, which required the use of urine and other malodorous substances in the process of treating the hides of cattle and other animals, had long been identified as one of the most polluting industries. As well as being forced to locate their workshops far from the centre of towns, tanners were amongst the principal targets of the sanitary legislation urban governments issued during times of plague.^20^

In his 1589 *First booke of cattell*, Leonard Mascall repeated Fitzherbert’s advice about separating infected cattle from the others, stating that ‘Thus must yee serue al your cattell that are infected or beeyng together in one pasture, so doing, ye shal auoyde the greater danger in this disease. For the murren is taken by venoumed grasse, by companie, and poisoned water, and by hunger’.^21^ In this context, seeking to understand the diseases which afflicted cattle was a worthy pursuit and was often undertaken by physicians, which further encouraged comparisons with human diseases. Writing at the time of the 1714 rinderpest outbreak in England, the physician Theophilius Lobb, in his study of the disease, wrote that Hippocrates ‘studied the Diseases of Beasts, as well as of Men, and Remedies against them…And thereby he set an Example worthy of Imitation’.^22^ When plague hit Provence nine years later, medical professionals and others would follow Hippocrates’ lead in comparing human plague and cattle plague to try and understand possible connections between the outbreaks.

## Human plague and cattle plague

The close appearance of plague in Provence after the cattle panzootic of the 1710s formed an instance of what Terence Ranger, in his study of cattle plague in late nineteenth-century Africa, termed ‘a coincidence of plagues of both beasts and men’, which created impressions of a ‘general…ecological crisis’ in the eyes of contemporaries.^23^ We can find the linking of the different types of disease and natural disasters, which Ranger observed for Africa, throughout the history of medieval and early modern Europe, with these associations becoming especially apparent during periods of outbreaks of plague in human populations. A range of people, from medical experts to government officials to members of the clergy, looked to the past and saw a pattern of plague striking both human and cattle populations concurrently, often combined with famine or crop failure to create the sense of a wider ecological crisis. In doing so, these writers were drawing on earlier ideas about the transference of disease between animals and humans.^24^ Cattle plagues and human plagues appeared to occur together. In 1376, the English Parliament heard of the plagues of men, murrains of cattle and ‘the failure of their corn and all other fruits of the earth’.^25^ A ‘great mortality of men and cattle’ struck Ireland in 1572 and again in 1648, which was also accompanied by famine.^26^ Looking to historic examples such as these helped make sense of contemporary crises. During the 1603 plague outbreak in England, the anonymous author of the *treatise A True bill of the whole number that hath died in the cittie of London* noted that during the reign of William the Conqueror (1066-87) there ‘fell so sore a plague, that the people died in such number that tillage decaied, and famine ensued, with rot of cattell, that men were faine to eate flesh of dogs, cats, & mise’.^27^ Writing in 1668, in the wake of the devastating 1665 plague outbreak and the following year’s Great Fire of London, the author of *Flagellum dei* also looked back to the compound crisis of the eleventh century for parallels with his own age, observing that during the reign of William the Conqueror there was a great fire in London which was followed by ‘a great mortality of Men and Cattel’.^28^

We also see an association between plagues in different species in sermons and other religious texts. The biblical encouragement to think in terms of graduated punishments helped shape the worldview of religious commentators, with the idea of intersecting or cascading disasters reflecting their ideas about societal decline.^29^ In his sermon on the plague in France in 1720, James Paterson looked back through history to give a range of examples of cattle and human plagues occurring together, such as that which struck Rome in 292 where ‘first the Cattle, then People were infected’, and in 1112 when ‘there was another Plague in England, and also a great Murrain of Cattle’.^30^ In 1631, the puritan preacher William Gough looked to another era of ecological crisis in the 1310s, which saw a major European-wide famine accompanied by a cattle panzootic.^31^ For puritans such as Gough, these apparently related calamities were a manifestation of God’s wrath – a view which also underpinned many plague tracts, even with the development of contagion theory. In his 1665 treatise, the Dutch-English physician Gideon Harvey (later physician to Charles II) wrote that: ‘Plagues do ordinarily survene great Inundations, Stinks of Rivers, unburied Carcases, Mortality of Cattel, Withering of Trees, Extinction of Plants, an extraordinary multiplication of Froggs, Toads, Mice, Flies, or other Insects and Reptils, a moist and moderate Winter, a warm and moist Spring and Summer, fiery Meteors, as falling Stars, Comets, fiery Pillars, Lightnings, *&c.* A ready putrefaction of Meats, speedy Moulding of Bread, briefness of the Small Pox and Measles, *&c’.* ^32^ In this view, cattle plagues were an element of a wider ecological turmoil which created the conditions for plague to infect humans. For the puritan minister Thomas Brookes, plague in humans and cattle was the same disease. In 1666, in the wake of the previous year’s devastating plague outbreak in England, Brookes, drawing on scripture (especially the books of Ezekiel and Exodus), wrote that the plague which had decimated the country in the previous year was ‘one and the same disease [as that which afflicted cattle], though when it is applied to cattel, it be usually rendred by murrain, yet when ‘tis applied to men, as in the Scripture last cited, it is commonly called the pestilence’.^33^ For Brookes, the pathology of the diseases was the same, and the differences were simply a matter of nomenclature.

For many, the appearance of animal murrains was a sure sign that human plague was to follow. In response to the Plague of Provence, in his 1721 plague tract, the physician George Pye stated that ‘Mortalities among the Cattle’ was one of the signs that an outbreak of plague in humans was about to occur.^34^ In the same year, Richard Brookes, also a physician, looked for historic examples of the same co-occurrence of cattle and human plagues that he saw happening at Provence. In his plague tract, Brookes noted that the devastating 1665 plague outbreak in London was ‘succeeded with Murrain of Cattle, and Scarcity of all sorts of Provisions’.^35^ Other medical writers seeking explanations for the outbreak in Provence looked to the past for similar examples of plague outbreaks following animal murrains. In his 1721 *Discourse concerning the Plague*, the anonymous author (who wrote under the name ‘Lover of Mankind’) drew on medical explanations of why human plague should follow cattle plague, noting how during an outbreak of plague in ancient Rome in 174 bce the disease ‘happened first among the cattle, which died in great numbers, that their carcasses putrified before they cou’d be remov’d. This infected the air of consequence, so that the spring following it broke out amongst the people in a terrible manner’.^36^ The author drew on the widely held miasma theory of the causes of disease, which held that diseases were caused by noxious air. For the ‘Lover of Mankind’, it was the unhealthy vapours released from the decaying corpses of infected animals that caused plague in humans. Yet the aetiology of cattle plague was a matter of debate in the discussions which re-emerged in the early 1720s in the wake of the issuing of the Quarantine Act in Britain.

## Cattle plague and the British Quarantine Act of 1721

Ascertaining the cause of cattle plague was of central importance in the early 1720s in Britain due to the restrictions the government wanted to place on trade with the continent to prevent the plague then spreading throughout southwestern France from entering the country. In 1710, Queen Anne’s government had passed a quarantine act intended to prevent infected ships coming from the Baltic, where an outbreak of plague had occurred, from entering Britain.^37^ There was a wider crisis in the Baltic in the 1710s as an epidemic of human plague overlapped with both an outbreak of cattle plague and famine conditions created by the Great Northern War. Following the appearance of the Plague of Provence, George I’s government reissued the 1710 Quarantine Act in August 1720. By the end of the year, the government sought to expand the scope of the quarantine provisions beyond those laid down in the 1710 act, as the plague continued to spread in France despite the extensive measures being taken to combat it there.^38^ To this end, the country’s leading physician, Richard Mead, son of the minister Matthew Mead mentioned earlier in the article, was tasked by the Lords Justices (George I was then in Hanover) with devising a plague treatise which laid out a scheme to prevent the disease from entering Britain. This resulted in his *Short Discourse Concerning Pestilential Contagion* (1720), which paved the way for the Quarantine Act of 25 January 1721 and its related measures. The key element in Mead’s plan was the enforcement of maritime quarantine for ships coming from infected places and for those in which sickness had been found – measures which were to remain in place for at least three years. The scale and duration of these methods led to significant opposition to the Act, which was seen by many as an example of excessive government power.^39^

In this context, cattle plague became a further front for debate between those who supported the introduction of quarantine measures and those who opposed them. The physician Richard Blackmore, who disputed the utility of the quarantine measures, in his plague tract of 1721, argued for the environmental – rather than contagionist – cause of disease. He stated that the wind carried plague in North Africa and that the disease was not spread by the import of infected goods from foreign countries.^40^ He wrote that the ‘Murrain among Cattle of different kinds is a Plague as contagious and destructive as the Pestilence among Men. Now ‘tis evident that the Murrain is bred at Home by unwholsome Herbs, bad Water, Malignant Air, etc, and is not deriv’d from Commerce maintain’d by Navigation with Foreign Countries; and the Parity of Reason will extend to the Plague, that affects Human Bodies, and then the Conclusion will be, that this great Disease is generally produc’d by Domestick Causes’.^41^ In other words, disease in both cattle and humans was caused by environmental factors, and thus the quarantine measures Mead was introducing on behalf of the government would not help prevent the spread of the plague.

Blackmore was not alone in employing the example of cattle plague to oppose the quarantine restrictions of the early 1720s. The unknown author of *Distinct Notions of Plague* (1722) also drew comparisons between human plague and cattle plague as a means to undermine the view that the disease spread through the import of foreign goods: ‘in all their Books of Physick [those of the ancient Greeks] we only hear, that Plague is a Fever; but no more of a Plague being carried and conveyed by Goods, than of the *Plagues* of *Cattle* being *bred* abroad, and brought into other Countries, by the like Conveyance’.^42^ As we see, the author looked to the ancients (from where ideas about miasma ultimately derived) to support his views that diseases of humans and animals were not spread through infected goods. Similarly, John Pringle in his 1722 plague tract wrote against the contagion-based measures then being used in France and said that ‘neither Goods nor Persons are capable of producing, or communicating the Plague’, but that it was due to the air. To support his views, Pringle gave the example of the recent cattle plague which spread from Central Europe across the continent to the Netherlands, ‘with the usual Velocity and Symptoms observed in Polish Plagues afflicting Mankind. But whatever may be allowed in favour of a Communicative Theory on the Continent, there can be no Salvo for its crossing the Channel to Islington [the location of London’s cattle market and the place where the first cases of cattle plague had been discovered in 1714]’.^43^ Likewise, the Scottish surgeon Peter Kennedy, in his 1721 plague tract, asserted that there was no known example of plague having ever entered the country from infected goods, ‘besides that, we very well know, that even Cattle and Beasts, when over Numerous together, with bad Air, Nourriture, Season, etc, are even also subject to pestilential Maladies, without ever having it sent box’d, pr pack’d up, to ‘em in Goods; and is often also thus communicated from them to Human Society’.^44^

These writers were reflecting longstanding views about the spread of disease, which were put forward by some of the most influential medical writers in early modern Europe, amongst whom there was a long tradition of considering cattle plague in comparison to plague in humans. Ambroise Paré, principal surgeon to four French kings in the latter half of the sixteenth century, wrote an important plague tract which was translated into English and remained influential into the eighteenth century. In this work, he considered how cattle became infected with plague. He stated that ‘vapours’, driven out of the earth by the alignment of the stars, ‘by their falling vpon Corne, Trees, and Grasse, infect and corrupt all things, which the Earth produces, and also killes those Creatures which feed vpon them’. That was why ‘skilfull Husbandmen, taught by long experience, neuer driue their Cattell or Sheepe to pasture, before that the Sunne by the force of his Beames, haue wasted and dissipated into Aire, this pestiferous dew hanging and abiding vpon boughes and leaues of Trees, Herbs, Corne, and Fruits’. For Paré, this miasma affected cattle first because their heads were closer to the ground, whereas he saw the plague which affected humans (and birds) as coming from above, ‘as those, who are neerer to Heauen’.^45^

This environmental explanation of the origins of disease (which we also find in the very earliest plague tracts, such as that produced by the Paris Medical Faculty in 1348) continued to be advocated in the late seventeenth and early eighteenth centuries amongst the leading medical practitioners in Europe.^46^ It was not a zero-sum game in terms of causation, and multiple explanations could sit alongside each other.^47^ In 1682, Dr. Wincler, physician to Prince Rupert of the Rhine, wrote to the Royal Society of London with an account of a recent cattle plague that had spread through Italy, Switzerland and Germany. He asserted that cattle were becoming infected through inhaling a ‘Blew-Mist, that fell upon those Pastures where the Cattle grazed, insomuch that herds have returned home sick’ and then died soon after. Wincler also believed that this disease could spread to humans, stating that ‘those persons that carelessly managed their Cattle without a due respect to their own health, were themselves infected and dyed away like their Beasts’. While reporting a rumour that the plague had been caused by three ‘Capuchin witches’ from Switzerland, he said that ‘it may more probably proceed from some Noxious Exhalations thrown out of the Earth’ by the recent earthquakes in that part of the world’.^48^

Nathaniel Hodges, who was a physician in London during the plague of 1665, wrote in great detail about cattle in his plague tract, which was republished in 1720 as a result of the plague in Provence. He saw all diseases as being caused by the noxious vapours found in unhealthy locations such as marshes.^49^ For Hodges, the release of these noxious vapours caused a general ecological crisis, which destroyed vegetation, created famine, and caused plague in both cattle and humans: ‘from which Adustion there may arise several Sorts of Distemperature; as Blasts upon Trees, and Diseases amongst Cattle; and at last end in Pestilence amongst Mankind’.^50^ Again, we see the view that environmental factors were the universal cause of disease in man, animal, and vegetation. For writers like Hodges, this explained why cattle and human plague occurred close together and were frequently accompanied by famine. These views of a general imbalance in nature resonated well with religious writers. In his 1721 sermon on the Plague of Provence and the threat it posed to Britain, the clergyman John Paterson, drawing on Virgil, stated that seasonal heats infected the grass, which then the animals ate, and this caused pestilence.^51^ As well as grass becoming affected through vapours, in their sermons on plague in 1720-21, clergymen such as David Jennings and Randolph Ford noted that God could stop the grass growing in order to kill cattle and cause famine, again producing a wider ecological crisis.^52^

## Contagion and cattle plague

A growing acceptance of ideas about contagion came to exist alongside existing traditional theories regarding the role of corrupt air in spreading disease. In his study of Ottoman cattle plagues, Sam White notes that ‘notions of contagion amongst animals were likely even older and more widely accepted than contagion amongst humans’, and we find such ideas as far back as antiquity.^53^ Contagionist understandings of cattle disease were well established in England by the early sixteenth century. Anthony Fitzherbert, in his 1534 treatise on cattle, highlighted ideas regarding contagion and cattle disease.^54^ These views were given a new impetus in the early eighteenth century following the publication of treatises on the cattle plague outbreak of the 1710s, particularly those by Bernardino Ramazzini, professor of the theory of medicine at the University of Modena, and Giovanni Maria Lancisi, the Italian physician and epidemiologist who also served as physician to three popes (Innocent XI, Innocent XII and Clement XI). Both men closely monitored the cattle plague in northern Italy and published influential works on the subject. Lancisi, who oversaw the campaign against the spread of the disease in Rome, believed it was a contagious disease which spread among cattle through their breath or on food. To minimise the risk of the disease spreading, he advocated the careful killing of diseased animals to contain their infected blood and stated that their carcasses were to be buried deeply and with nothing removed from them (advice that we also saw already in the English veterinary manuals of the sixteenth century). Cattle sheds should be fumigated, as should any items of clothing which had been near the animals. Lancisi cautioned against consuming meat from infected carcasses or milk from infected animals, though he stated that he did not know if the disease could be spread this way. His main concern was that other infections (such as ‘cruel diarrhoea’) – rather than cattle plague – could spread to humans who ate the meat from these animals.^55^ Nonetheless, he allowed for interspecies infection in that humans could spread it to healthy cattle, as could other animals such as birds.^56^

Like Lancisi, Ramazzini studied first-hand the outbreak of cattle plague and examined cattle that died from the disease.^57^ He argued against a universal source of infection in the air or the fields, stating that it was a disease particular to cattle and spread ‘through the secretions and the excreta of ailing and dead oxen’, infection lingering in places where infected animals had been kept, and through infected hides.^58^ The treatments he recommended for use on sick cattle were similar to those used to treat humans infected by plague, including: bleeding, fumigation and medicines. He thought that cattle plague did not afflict humans, but that they could be carriers of the disease and spread it amongst healthy cattle. Ramazzini’s views were especially influential in England in the early eighteenth century, probably because they appeared in *Philosophical Transactions of the Royal Society* in 1714.^59^ Richard Mead and Richard Bradley (see below), who wrote extensively about animal murrains in their plague treatises of the early 1720s, were fellows of the Royal Society. Indeed, Ramazzini’s findings were probably familiar to many of the other people who wrote about the Plague of Provence, which may further explain the prominence of discussions of cattle plague in the tracts of this period. Certainly, the outbreak of plague in France led to a resurgence in interest in cattle plague at the Royal Society. For instance, a further report on the recent cattle murrain was presented to the Royal Society in 1720, soon after it became clear that plague was infecting Provence, where the author described the disease and outlined its symptoms.^60^

Richard Bradley – who would be appointed the first professor of Botany at the University of Cambridge just after the Plague of Provence – drew on Ramazzini’s work, especially his remarks on the physiology of cattle and how they became infected with disease, in his 1720 *Plague at Marseilles Considered*, a popular work which was already on its third edition by 1721. Bradley’s particular interest in cattle was probably a result of his belief in a unified view of infectious diseases in people, animals, and plants. In this plague treatise, Bradley looked to the outbreaks of cattle plague which had occurred in the 1680s and 1710s, both of which, as we saw, were discussed by the Royal Society. Taking the similarities advocated for the treatment of humans and cows as his starting point, Bradley argued that the cattle plague, which spread to England from the continent, was infectious and derived from the contaminated breath of sick animals. He also wrote that it was spread by ‘unwholesome standing water, where ‘tis probable some poisonous insects were lodged and bred’, because of a long, hot and dry summer with constant winds from the east. Yet this was not simply a further iteration of the miasmatic ideas regarding infection, which others had advanced. Rather, the ‘insects’ he discussed were invisible to the naked eye and caused disease in animals and humans, as well as blights in crops. Ideas about invisible ‘insects’ causing infection, which were already present in some texts on plague from the late Middle Ages, were gaining more currency during the sixteenth century. The most famous early advocate of contagion theory was Girolamo Fracastoro and his ideas about ‘seeds of contagion’, often seen as germ theory *avant la lettre*, to which technological advances in microscopes in the seventeenth century lent greater credence.^61^ During the Roman plague of 1656–7, Anthanius Kircher had used microscopes to study the blood of the infected and claimed that microscopic organisms (‘animalcules’) were responsible for causing the disease, effectively outlining the principle of animate contagion.^62^ These views surfaced again during the Plague of Provence. In the preface to the popular *Observations faites sur la peste*, published in 1721 by Jean Baptiste Bertrand, Jean Baptiste Goiffron (the medical botanist who served in Lyon’s health board) also argued that plague was spread by invisible insects, while in his 1722 work, the Dutch mathematician and physicist Nicolaas Hartsoeker proposed that plague was caused by microscopic insects biting humans.^63^

During the prolonged cattle plague outbreaks which occurred from the 1740s, various writers drew on the ideas of Hartsoeker and Antonie van Leeuwenhoek to argue that invisible insects entered the bodies of cattle when they breathed in and attacked their intestines.^64^ Yet in his 1720 treatise on the Plague of Provence, Bradley, who was also an advocate of the use of microscopes, was already comparing cattle bodies to human bodies and positing that they too could be infected by insects brought in via food. He noted that cattle plague was a contagious disease and that sick animals soon infected the healthy. Bradley averred that any uninfected animals brought into fields where infected animals had spent the summer, or else warm sheds where they had been placed, became infected themselves, though he stated that frost killed the infection in the fields over the winter. This drew on Ramazzini’s idea that the ‘effluvia’ found in sick cattle and their environment infected others. Bradley also considered the possibility of the transmission of disease from humans to animals, whereby infected clothing worn by cowherds infected healthy cattle.^65^ Bradley and Ramazzini’s views that humans, while not becoming infected with cattle plague themselves, could unwittingly spread it to animals were shared by others. Thomas Bates, George I’s surgeon, who led the campaign against rinderpest in 1714, in his account of the panzootic wrote that while diseases rarely spread between species, they could infect members of the same species through materials such as clothing, ‘to which the Infectious Effluvia of the Diseased had adhered’. In addition, he believed that ‘more Hundreds died from the Infection, which was carried by the Intercourse that the Cow keepers had with each other, than single ones by the original Putrification’.^66^

This logic was also extended to the wider environment, even by writers who were opposed to contagionist methods. The anonymous author of the 1721 plague tract *A new discovery of the nature of plague*, which was written against Mead and others of their view, notes that ‘Places and Things’ could be infected with diseases. To support this assertion, he cited the cattle plague which affected England in the 1710s stating that this ‘Distemper among the Black Cattle [was] very destructive; and when every Body thought it was abated, and gone (the Cattle being well for eight or ten Months), it broke out a-new, which was occasioned by putting Cattle in those Houses and Places, where the Infected were lodged the Season before; nor could this be cured, till that the Timber, and other Things in the Houses were washed with Soap, Lees, and other searching and penetrating Liquors; and the Parts smoked, which killed the Vermine’.^67^ Theophilius Lobb wrote in 1711 that cattle plague ‘is not produced by any poisonous, coagulating Particles of matter diffused in the Air, and received with Breath into their Bodies’, nor by any poison in the food or water they consume, but that it was caused by an infection which spread amongst cattle, similar to plague in humans, through ‘infectious particles, some of which, emitted from the Body of the first sick Beast, is conveyed by Contact, or somehow, thro’ the Air, into the Bodies of the Cattle infected by it, and so on’. The treatment he prescribed was essentially that used to treat humans during plague. Cattle herds were to be closely monitored, and at the first sign of sickness in an animal, they were to be segregated and brought to a separate enclosure at a distance from healthy animals, where they were to receive treatments and medicines. Those who were to tend the beasts were to wear protective clothing and use vinegar to disinfect their hands and ‘keep as much as possible to the Windward-Side of them, and carefully avoid Taking their Breath’.^68^ As well as considering how cattle became infected, these writers considered the extent to which animals could transmit diseases to humans and make them sick.

## Cattle as vectors of disease in humans

By the early seventeenth century, medical writers were already conceiving of separate diseases for different species. In his 1604 plague tract, the physician Francis Herring wrote that not all illnesses were alike and that different illnesses infected different species. ‘Concerning the opinion it is not true, that euery venome is like to Arsenicke, neither that euery poison is like in substance and Nature with another poison: neither can all contagious seminaries be like to Arsenicke, since they are not of the same violence, Analogie, or similitude one with another, as might be easily shewed some infecting onely *Cattle*, others Fishes, others men’.^69^ Rather than being one disease which infected all, diseases were often specific to individual species. The growing purchase of such views in the early eighteenth century led some medical experts to declare that the Plague of Provence was unrelated to the cattle plague which preceded it. In 1720, Richard Mead wrote that ‘all *Plagues* do not indifferently affect all Kinds of living Creatures; on the contrary, most are confined to a particular *Species* of them; like the Disease of the *Black Cattle* a few Years since, which neither proved Infectious to other Brutes, nor to Men’.^70^ While the author of *A new discovery of the nature of plague* opposed Mead’s quarantine measures and contagionist views, he nonetheless agreed with him that there were generally separate diseases for separate species: that ‘the different Species of Beasts have sudden Distempers, which sweep away whole Droves at once, and we find often, that what rots the Sheep, has no influence on the black Cattle’, although both this writer and Mead did allow that there could be wider diseases which affected all.^71^

While downplaying the direct transmission of disease from cattle to humans, Mead himself declared in a later edition of his plague tract (and possibly in response to the views of men such as Brookes and Pye, which we saw above) that human plague could be spread to other animals. He stated that: ‘tho’ I am well aware that there are Plagues among Animals, which do not indifferently affect all kinds of them, some being confined to a particular Species (like the Disease of the Black Cattle here, a few Years since, which neither proved infectious to other Brutes, not to Men;) yet it has always been observed that the true Plague among Men has been destructive to all Creatures of what kind soever’. To support this claim, Mead stated that birds had become infected by infected goods on a ship brought from Cairo in 1726, which killed men and birds exposed to the disease alike.^72^ As a leading proponent of maritime quarantine, this claim probably formed an element in his assertion on the use of quarantine on goods coming from the continent in the wake of the Plague of Provence by raising the threat that if it entered Britain that it could decimate animal and human populations.

Some physicians writing about the Plague of Provence sought to provide an account which argued for separate diseases between species but also allowed for humans to be carriers of bovine disease (but which did not cause them to become sick). In his plague tract of 1721, Philip Rose, a member of the Royal College of Physicians, noted that the cattle plague outbreak of the 1680s did not affect human populations. Drawing on Hippocrates, he stated that disease in both man and beast was caused by ‘Air Distempers’, having too much or too little of it, ‘of filled up with unwholsome Particles, it penetrateth into the Body’. While echoing the statements we saw above by Harvel in the seventeenth century in stating that animal and human bodies were both infected by corrupt air, Rose declared the physiological differences between them produced separate diseases: ‘as the Bodies of one Species differ from another, so likewise they differ in Nature, and by their different Food, they must differ in their Constitution…Therefore when the Air is impregnated and vitiated with such Effluviums, as are contrary to Human Nature, then Mankind is the Subject of the Disease. And when the Air’s Constitution is such, as to be unnatural to some one Species of Animals, then that very Species is obnoxious to the Disease’.^73^ Yet Rose allowed for the possibility of an interspecies infection between cattle and humans: ‘a Sickness amongst Cattle is transferable to the humane Species, hath not yet appeared on any good Foundation; but to remove this Difficulty, no one doubts but that a Plague amongst Cattle, from some common Cause, as a Corruption of the aerial Nitre, and which differs from a Plague amongst Men but in Degree, may also be transmitted to the humane Species’, but in a weaker form and not one which could make humans sick.^74^

In terms of allowing provisions into towns, Rose asked, ‘Can you be sure that the Men that drive the Cattle, and bring you every Thing else, for the support of Life, are not infected? I am sure no rational and serious Man can say, they are not, nor the Cattle also’.^75^ Rose, who we saw believed in the environmental role of transmission of disease and the role of miasmas, was quite possibly considering that cattle could produce the conditions necessary for the spread of disease. Certainly, cattle occupied an ambiguous position with regards to plague in humans. On one hand, the consumption of good-quality red meat was, by the sixteenth century, an important element in a medically approved diet for the plague-infected.^76^ The author of the 1721 tract *Medicina Flagellata* even claimed that populations in the Far East were more susceptible to plague than Europeans because of their ‘low and pitiful Fare’, whereas Europeans were more robust because ‘by feeding on good Flesh, and drinking of strong generous Wine, which secures them from the Power of that Malignancy’.^77^ The need for red meat during times of plague encouraged the movement of large numbers of cattle to cities. This meant that during times of plague, large quantities of meat needed to be imported into towns and cities. Pichatty de Croissantte, a member of the municipal council of Marseille in 1720, notes in his journal that large amounts of cattle (as well as sheep and corn) were brought to the stricken city.^78^ Plague regulations on the movement of people and goods could hinder this process. In 1626, the author of the *Lachrymæ Londinenses* complained about the corruption and unjust actions of the justices of the peace in the counties surrounding London during the previous plague, particularly that ‘they hindred our *London* Butchers to come into their adjacent Townes and Hamlets to fetch Cattell, for our food’.^79^

Yet while the consumption of good-quality meat could help prevent plague, decaying meat was seen as one of the causes of diseases, again relating to medical ideas about miasma and infection through foul air. In his 1671 influential work *Loimologia* (which was republished in 1720 in response to the Plague of Provence), Nathaniel Hodges – who was praised for his work during the 1665 plague of London and was admitted to the Royal College of Physicians in 1672 on the basis of the advice he included in this work – observed that before Cardinal Wolsey’s plague reforms of 1518 ‘they used to kill all Manner of Cattle within the Walls, and suffer their Dung and Offals to lie in the Streets’ which helped create the ‘Malignant Vapours’ which caused the disease.^80^ *A new discovery of the nature of plague* also notes that London’s ‘Air is thick and gros, from the great Resort of People, and from the great Slaughter of Cattle’.^81^ Such concerns had long been present in London, the population of which consumed a large amount of meat. In the early fifteenth century, the stench from the heaps of manure at Smithfield cattle market caused ‘right grevious nusiance’.^82^ By the end of the sixteenth century, the rapidly expanding city of London became a massive consumer of meat and surrounding areas such as Romney Marsh were turned into grazing grounds for cattle brought into the city for slaughter. We find similar developments on the continent in the rearing of cattle specifically for slaughter.^83^ Like the poor, who were also believed to be a cause of the disease, which they spread through their corrupt breath, animals were the target of legislation designed to remove them from public spaces.^84^

Beyond this concern with noxious smells from cattle, there was also a concern that the consumption of the flesh from diseased animals caused plague in humans, especially with regard to the poorer classes. In *Medicina Flagellata* (1666), the author observed that just before the 1665 plague ‘there was a great Mortality amongst the Cattel from a very wet Autumn, and their Carcasses being sold amongst the ordinary People at a very mean Price, a great many putred Humours might proceed from thence; and this, in the Opinion of many, was the Source of our late Calamities, when it was observed this fatal Destroyer raged with greater Triumph over the common People’.^85^ The eating of infected meat by the poor during these periods of crisis is recorded in other sources and eras.^86^ As we saw above, Lancisi recommended that people not eat animal products in case it was possible for the disease to be transmitted that way. Nonetheless, as in other aspects of links between the two diseases, the claim that eating infected cattle meat led to plague was debated. Nathaniel Hodges wrote on the opinion of many that the eating of ‘corrupted, or rotten Flesh’ was a cause of the plague. He noted that ‘the Year before the late pestilential Sickness, there was a great Mortality amongst the Cattel, from a very wet Autumn, whereby their Carcases were sold amongst the ordinary People at a very mean Price; and a great deal of putrid Humours in all likelihood produced from thence’. Though he does not agree with this, saying that it could lead to ‘epidemical Diseases, but not a true Pestilence’.^87^

During the early modern period, there was an increasing distinction placed on different forms of animals, particularly between vermin such as mice or birds, which stole food and spread disease, and the domesticated animals that were useful to humans.^88^ Yet this distinction could be blurred when it came to cattle, as they were one of the most important beasts for human society, but also seen as a cause for disease. Such views persisted into the nineteenth century when they could be employed in a colonial context to reflect racist views about non-Europeans. An official report on the outbreak of plague in the Kumaon region in northern India in the 1850s found ‘On the ground-floor herd the cattle; in this compartment the dung is allowed to accumulate till such time as there is no room left for the cattle to stand erect; it is then removed and carefully packed close around all sides, so that the house literally stands in the centre of a hot-bed…. In many instances we have seen it accumulated above the level of the floor of the upper story in which the family lives’. In his report of the irruption of typhus in the Yusufzai valley at Peshawar in 1852, the doctor stated that ‘it arose mostly in the filthy Mohammedan houses, shared by cattle and human beings’.^89^ Indeed, the link between cattle disease and plague in humans persisted into the modern day, with Graham Twigg (in a widely discredited theory) claiming the plague was really pulmonary anthrax spread to humans from cattle who, in the later Middle Ages, were living in greater proximity to them than before.^90^

## Conclusion

This article shows how the outbreak of plague in Provence in 1720 led to a re-emergence of long-standing fears about the cross-species transmission of disease. We saw how a range of writers from medical doctors to clergymen in the 1720s identified co-occurrences of plague in cattle and plague in humans and sought to understand the causal factors which produced this recurring pattern – factors which were typically located in contemporary environmental, societal and medical understandings of the causes of disease. In the early eighteenth century, people of different backgrounds, opinions and outlooks considered the nature of human and animal diseases. More broadly, like the African societies Terence Ranger studied, wider cultural and societal factors, such as religious beliefs, also provided an interpretative framework for early modern populations to understand ‘coincidence[s] of plagues of both beasts and men’.^91^ According to Bernardino Ramazzini, who argued against the cross-species transmission of disease, adverse climatic conditions could produce diseases in both animals and humans, which, so he argued, explained why animal plagues often preceded human plagues and thus acted as a warning sign.^92^ As we saw, Ramazzini’s views were influential amongst men such as Mead and Bradley in the contagionist debates which occurred during the Plague of Provence.

While focusing on the Plague of Provence, this article has also shown that the debates around co-concurrent outbreaks of contagious diseases amongst animal and human populations, including fears that diseases jumped from one species to another, were not a product of the eighteenth century. Rather, the fierceness of the outbreak of plague which struck Provence in the 1720s – and the fear that it would spread across Europe, as previous plagues had done – caused a re-emergence of much older fears about the cross-species transmission of disease. Yet, unlike their predecessors, those writing about the Plague of Provence in the 1720s could draw on new technologies (such as microscopes) and intellectual ideas (such as that of animate contagion) to understand the interplay between the diseases. Nonetheless, if the intellectual and scientific developments of the early Enlightenment could be harnessed to better under the causes of plague and murrains, the methods devised to combat outbreaks of disease amongst domesticated animals in the eighteenth century, such as the sequestration of livestock, the banning of fairs and the introduction of quarantine controls, were ultimately derived from the longstanding measures which had been developed from the fourteenth century to combat plague. If murrains and plague were ultimately separate diseases, as many of those medical writers of the 1720s had argued, in some ways at least, animals and humans had a shared experience of contagious disease.

## Footnotes

1 It was once falsely believed that the plague of Provence was the last major outbreak of plague to strike Europe (and this claim occasionally resurfaces) though there were major later outbreaks, such as those at Messina in 1743, which killed 48,000 people, and Moscow in 1770–1, when more than 100,000 died. It is not fully understood when the second pandemic of plague ended, though it was likely still present in North Africa as late as the 1870s: Samuel K. Cohn , ‘Epidemiology of the Black Death and Successive Waves of Plague’, Medical History 52 (2008), 74 PMC263003518575083.

2 See especially: Henri Alezais , Le blocus de Marseille pendant la peste de 1720 (Valence: Valentinoise, 1907); René Bruni , Le pays d’Apt malade de la peste (Aix-en-Provence: Édisud, 1980); Gilbert Buti , Colère de Dieu, mémoire des hommes. La peste en Provence 1720-2020 (Paris: Éditions du Cerf, 2020); idem, *La peste à Valette. La peste au village, 1720-21* (Marseille: Éditions Autres Temps, 1998); Charles Carrière et al, Marseille, ville morte. La peste de 1720 (Paris: M. Garçon, 1968); Odile Cayeux , Arles et la peste, 1720-21 (Aix-en-Provence: Presses universitaires de Provence, 2009); Pierre Chauvet , La lutte contre une épidémie au 18e siècle. La peste de Gévaudan (1720-23) (Paris: A. Legrand, 1939); Sébastien Demichel , Risque et vigilance sanitaire: la côte méditerranéene française face à la peste, 1700-1750 (Berlin: De Gruyter, 2024); Cindy Ermus , The Great Plague Scare of 1720: Disaster and Diplomacy in the Eighteenth-Century Atlantic World (Cambridge: Cambridge University Press, 2023); Paul Gaffarel and M. de Duranty , La Peste de 1720 à Marseille et en France d’après des documents inédits (Paris: Perrin, 1911); Michel Goury , Un homme, une navire. La peste de 1720 (Marseille: Laffitte, 2013); Armand Praviel , Belsunce et la peste de Marseille (Paris: Éditions Spes, 1936); Junko Takeda , Between Crown and Commerce: Marseille and the Early Modern Mediterranean (Baltimore: Johns Hopkins University Press, 2011). See also: Fleur Beauvieux, ‘Expériences ordinaires de la peste. La société Marseillaise en temps d’épidémie (1720-1724)’ (unpublished PhD thesis: École des Hautes Études en Sciences Sociales, 2 vols); Claude Bollet, ‘A propos du traitement de la peste en 1720 à Marseille’ (unpublished PhD thesis: Université de Marseille, 1983); Jamel El Hadj, ‘Les chirurgiens et l’organisation sanitaire contre la peste à Marseille, 17e-18e siècles’ (unpublished PhD thesis: École des Hautes Études, 2014); Françoise Hildesheimer , Le Bureau de Santé de Marseille sous l’ancien régime (Marseille: Fédération Historique du Provence, 1980). Jean-Noël Biraben’s two volume *Les hommes et la peste en France et dans les pays européens et méditerranéens* (Paris: Mouton, 1975) also focuses heavily on the plague of Provence.

3

Carole Rawcliffe , “The Abominable Offence and Poisoning of the Air”: Animals, Miasmas, and Urban Epidemics in Late Medieval Britain’, in Axel C. Hüntelmann et al (eds), Animals and Epidemics: Interspecies Entanglements in Historical Perspective (Cologne: Böhlau Verlag, 2024), 53–70 ; idem, *Urban Bodies: Communal Health in Late Medieval English Towns and Cities* (Woodbridge: Boydell, 2013), 153–63; William Riguelle, ‘Purifying the Streets: Legislation Against Animals During Epidemics in the Towns of the Southern Netherlands and the Principality of Liege, 1600-1670’, in Hüntelmann et al (eds), *Animals and Epidemics*, 71–84.

4 In contrast to the literature on cattle plagues cited below, the historiography of plague in humans is too vast to include here. For some prominent recent studies, see: Samuel K. Cohn , Cultures of Plague: Medical Thinking at the End of the Renaissance (Cambridge: Cambridge University Press, 2009); Ermus, *Great Plague Scare*; John Henderson , Florence Under Siege: Surviving Plague in an Early Modern City (New Haven: Yale University Press, 2019); Ruth Mackay , Life in a Time of Pestilence: The Great Castilian Plague of 1596-1601 (Cambridge: Cambridge University Press, 2019); Neil Murphy , Plague, Towns and Monarchy in Early Modern France (Cambridge: Cambridge University Press, 2024).

5 It is worth noting that while recent genetic studies of bodies found in plague pits have identified the presence of *Yersinia pestis* (the bacterium of the third pandemic), the connection between rats and the second pandemic of plague remains contested. For these debates, see: Cohn, ‘Epidemiology of the Black Death’, 74–100; Lester K. Little , ‘Plague Historians in Lab Coats’, Past & Present, 213 (2011), 267–9022442843 10.1093/pastj/gtr014.

6

Ian Kershaw , ‘The Great Famine and Agrarian Crisis in England 1315-1322’, Past & Present, 59, 1, (1973), 1–50 ; Timothy P. Newfield , ‘A Cattle Panzootic in Early Fourteenth-Century Europe’, Agricultural History Review, 57, 2, (2009), 155–90; Philip Slavin , ‘The Fifth Rider of the Apocalypse: The Cattle Plague in England and Wales and its Economic Consequences, 1319-1350’, in Simonetta Cavaciocchi (ed.), Le interazioni fra economia e ambiente biologico nell’Europa preindustriale secc. XIII-XVIII (Florence: University of Florence Press, 2010), 165–79; idem, ‘The Great Bovine Pestilence and Its Economic Consequences in England and Wales, 1318-1350’, *Economic History Review*, 65, 4 (2012), 1239–66. See also: Timothy P. Newfield , ‘Livestock Plagues in Late Antiquity, with a Disassembling of the Bovine Panzootic of A.D. 376-386’, Journal of Roman Archaeology, 30 (2017), 490–508 ; idem, ‘Domesticates, Disease and Climate in Early Post-Classical Europe: The Cattle Plague of c.940 and its Environmental Context’, *Post-Classical Archaeologies*, 5 (2015), 95–126; idem, ‘A Great Carolingian Panzootic: The Probable Extent, Diagnosis and Impact of an Early Ninth Century Cattle Pestilence’, *Argos*, 46 (2012), 200–10; idem, ‘Human-Bovine Plagues in the Early Middle Ages’, *Journal of Interdisciplinary History*, 46, 1 (2015), 1–38.

7

Karl Appuhn , ‘Ecologies of Beef: Eighteenth-Century Epizootics and the Environmental History of Early Modern Europe’, Environmental History, 15, 2 (2010), 268–87; Dorothee Brantz , ‘Risky Business’: Disease, Disaster and the Unintended Consequences of Epizootics in Eighteenth- and Nineteenth-Century France and Germany’, Environment and History, 17, 1 (2011), 35–51 ; Reinhold Dorwart , ‘Cattle Disease (Rinderpest?): Prevention and Cure in Brandenburg, 1665-1732’, Agricultural History, 33, 2 (1959), 79–85 ; Arvel B. Erickson , ‘The Cattle Plague in England, 1865-1867’, Agricultural History, 35, 2 (1961), 94–103 ; J. A. Faber , ‘Cattle-Plague in the Netherlands During the Eighteenth Century’, Mededelingen van de Landbouwhogeschool te Wageningen, 62, 11 (1962), 1–7 ; John Fisher , ‘To kill or not to kill: the eradication of contagious bovine pleuro-pneumonia in western Europe’, Medical History, 47, 3 (2003), 314–3112905917 PMC1044631; idem, ‘The Economic Effects of Cattle Disease in Britain and its Containment, 1850-1900’, *Agricultural History*, 54, 2 (1980), 278–94; Sherwin A. Hall , ‘The Cattle Plague of 1865’, British Veterinary Journal 122, 6 (1966), 259–64; C. Huygelen , ‘The immunization of cattle against rinderpest in eighteenth-century Europe’, Medical History, 41, 2 (1997), 182–969156464 10.1017/s0025727300062372PMC1043905; Dominik Hunniger , ‘Policing Epizootics: Legislation and Administration During Outbreaks of Cattle Plague in Eighteenth-Century Northern Germany as Continuous Crisis Management’, in Karen Brown and Daniel Gilfoyle (eds.), Healing the Herds: Disease, Livestock Economies and the Globalization of Veterinary Medicine (Athens, Ohio: Ohio University Press, 2010), 92–107 ; idem, *Die Viehseuche von 1744-52. Deutungen und Herrschaftspraxis in Krisenzeiten* (Neumünster: Wachholtz, 2011); idem ‘Bugs, Worms and Dying Cattle: Multispecies Histories of Cattle Plague Outbreaks in the Long Eighteenth Century’, in Hüntelmann et al (eds.), *Animals and Epidemics*, 85–94; Peter A. Koolmees, ‘Epizootic Diseases in the Netherlands, 1713-2002’, in Brown and Daniel Gilfoyle, *Healing the Herds*, 19–41; Filip van Roosbroeck , ‘Caring for Cows in a Time of Rinderpest. Non-academic Veterinary Practitioners in the County of Flanders, 1769-1785’, Social History of Medicine 32:3 (2019), 502–22; idem, ‘To cure is to kill? State intervention, cattle plague and veterinary knowledge in the Austrian Netherlands, 1769-85’ (unpublished PhD thesis: University of Antwerp, 2016); Reinhard Stahle, ‘Verlauf und Verbreitung der Rinderpest in Herzogtum Bremen und Verden von 1715 bis 1781’ (unpublished PhD thesis: Tierärztliche Hochschule, 1981); Carsten Stühring , Der Seuche begegnen. Deutung und Bewältigung von Rinderseuchen im Kurfürstentum Bayern des 18. Jahrhunderts (Frankfurt am Main: Peter Lang, 2011); Carsten Stuhring, ‘Managing Epizootic Diseases in 18^th^-Century Bavaria’, in Cavaciocchi, *Interazioni*, 473–80; Adam Sundberg , Natural Disaster at the Closing of the Dutch Golden Age: Floods, Worms and Cattle Plague (Cambridge: Cambridge University Press, 2022), 51–88, 212–50; François Vallet , Les boeufs malades de la Peste: la Peste Bovine en France et en Europe XVIIIe-XIXe siècle (Rennes: Presses universitaires de Rennes, 2009); Johanna Widenberg , Den stora kreatursdoden. Kampen mot boskapspest och mjaltbrand i 1700-talets svenska rike (Stockholm: Carlsson, 2017); idem, ‘Cattle Plague and Society. Rinderpest, Anthrax and Epizootic Control in the Eighteenth Century Kingdom of Sweden’, *1700-tal. Nordic Journal for Eighteenth-Century Studies*, 17 (2020), 8–33; Lise Wilkinson , ‘Rinderpest and the Mainstream Infectious Disease Concepts in the Eighteenth Century’, Medical History, 28 (1984), 129–506387340 10.1017/s0025727300035687PMC1139420. For a rare examination of the links between epizootics and epidemics in Enlightenment Europe, though from a literary perspective, see: Lucinda Cole, ‘What is an Animal? Contagion and Being Human in a Multispecies World’, *Lumen*, 40 (2021), 35–53. While research is lacking on European cattle plagues of the fifteenth, sixteenth and seventeenth centuries, those in Asia have received some scholarly attention, such as that which ravaged Anatolia in the 1590s and those in seventeenth-century Japan and Korea: Hiroshi Kishi, ‘A Historical Study on Outbreaks of Rinderpest during the Yedo Era in Japan’, *The Yamaguchi Journal of Veterinary Medicine*, 3 (1976); Dong Jin Kim , Han Sang Yoo and Hang Lee , ‘Effects of the Periodical Spread of Rinderpest on Famine, Epidemic, and Tiger Disasters in the Late 17^th^ Century’, Korean Journal of Medical History, 23 (2014), 1–56 24804681 10.13081/kjmh.2014.23.1PMC10565085; Sam White , ‘A Model Disaster: From the Great Ottoman Panzootic to the Cattle Plagues of Early Modern Europe’, in Nükhet Varlik (ed.), Plague and Contagion in the Islamic Mediterranean (Amsterdam: Amsterdam University Press, 2017), 91–116 .

8 In addition to the specific studies of cattle plagues in the fourteenth and eighteenth century cited in the previous two footnotes, some wider studies of animal diseases include discussions of medieval and early modern cattle plagues. See: Jean-Jacques Paulet , Recherches historiques et physiques sur les maladiés epizootiques avec les moyen d’y remédier dans tous les cas (Paris: Ruault, 1775); Georges Curasson , La Peste Bovine (Paris: Vigot, 1932); Wilhelm Dieckerhoff , Geschichte der Rinderpest und ihrer Literatur. Beitrag zur Geschichte der vergleichenden Pathologie (Berlin: Enslin, 1890); George Fleming , Animal Plagues: Their History, Nature, and Prevention (London: Champman and Hall, 1871); George A. Spinage, *Cattle Plague: A History* (New York: Kluwer/Plenum, 2003). For criticisms of these and similar works, which were often done by mycologists or veterinary specialists rather than historians, see: Timothy P. Newfield, ‘Premodern European Animal Plagues. Common but Enigmatic’, in Hüntelmann, *Animals and Epidemics*, 236–7.

9

John Booker , Maritime Quarantine: The British Experience, c.1650-1900 (Farnham: Routledge, 2007), chapter 4; Ermus, *Great Plague Scare*, chapter 3; Margaret DeLacy , The Germ of an Idea: Contagionism, Religion and Society in Britain 1660-1730 (New York: Palgrave, 2016), chapter nine; Arnold Zuckerman , ‘Plague and Contagion in Eighteenth-Century England: The Role of Richard Mead’, Bulletin of the History of Medicine, 78, 2 (2004), 273–308 15211050 10.1353/bhm.2004.0105; Charles Mullett , The Bubonic Plague and England: An Essay on the History of Preventative Medicine (Lexington, 1956), 275–82; idem, ‘The English Plague Scare of 1720-23’, *Osiris*, 2 (1936), 484–516; Paul Slack , ‘Perceptions of Plague in Eighteenth-Century Europe’, Economic History Review, 75 (2022), 138–56.

10 In the pre-modern era, the term ‘cattle’ could be used a collective term for livestock (including animals such as sheep and horses, as well as cows). Yet contextual information suggests that when writing of cattle plagues in the later medieval and early modern eras, contemporaries were using the term in the modern sense of the word to refer to cows, calves, etc, though it may also be the case that at other times writers were using the term to refer to wider murrains in different species of domesticated animals, such as sheep which were also prone to epizootics. For early modern definitions of ‘cattle’, see the relevant entry in the *Oxford English Dictionary.*

11 For plague tracts, see: John Aberth , Doctoring the Black Death: Europe’s Late Medieval Medical Response to Epidemic Disease (London: Rowman & Littlefield, 2021); Cohn, *Cultures of Plague*; Joël Coste , Représentations et comportements en temps d’épidémie dans la littérature imprimée de peste, 1490-1725: contribution à l’histoire culturelle de la peste en France à l’époque moderne (Paris: Honoré Champion, 2007); Lori Jones , Patterns of Plague: Changing Ideas about Plague in England and France, 1348-1750 (Montreal: McGill-Queen’s University Press, 2022).

12

Keith Thomas , Man and the Natural World: Changing Attitudes in England (London: Penguin, 1983), 25 .

13

Karen Raber , ‘From Sheep to Meat, From Pets to People: Animal Domestication 1600-1800’, in Matthew Senior (ed.), A Cultural History of Animals in the Age of Enlightenment (London: Bloomsbury, 2009), 77 ; Peter Edwards , ‘Domesticated Animals in Renaissance Europe’, in Bruce Boehrer (ed.), A Cultural History of Animals in the Renaissance (London: Bloomsbury, 2009), 77 ; Thomas, *Natural World*, 94, 118–19.

14
*A godly ballad of the just man Job. Wherein his great patience he doth declare, his plagues and miseries, and yet did not despair* (London: F. Coles, J. VVright, Tho, Vere, and VV. Gilbertson, 1658).

15
*Orders, thought meete by her Maiestie, and her priuie Councell, to be executed throughout the counties of this realme, in such townes, villages, and other places, as are, or may be hereafter infected with the plague* (London: Christopher Barker, 1578), 8. These orders were reissued by subsequent monarchs during times of plague: *Orders formerly conceived and agreed to be published by the Lord Major and the aldermen of the City of London: and the justices of peace of the counties of Middlesex and Surrey, concerning the infection of the plague. And now re-printed and published by order of the Honourable House of Commons* (London: Richard Coates, 1646); *Royal College of Physicians, Certain necessary directions, aswell for the cure of the plague as for preuenting the infection; with many easie medicines of small charge, very profitable to His Maiesties subiects* (London: John Bill & Christopher Barker, 1636), 52.

16 Matthew Mead, *Solomon’s prescription for the removal of the pestilence, or, The discovery of the plague of our hearts, in order to the healing of that in our flesh* (London: [n.p], 1665), 27.

17

George Wither , Britain’s remembrancer containing a narration of the plague lately past; a declaration of the mischiefs present; and a prediction of iudgments to come; (if repentance prevent not) (London: John Grismond, 1628), 121 (see also: 163).

18 Wilkinson, ‘Rinderpest’, 129, 131.

19

Walter W. Skeat (ed.), The Book of Husbandry by Master Fitzherbert (London: Trübner & Co, 1882), 79 ; Cole, ‘Contagion’, 37.

20 Rawcliffe, *Urban Bodies*, 207–8.

21

Leonard Mascall , The first booke of cattell wherein is shewed the gouernment of oxen, kine, calues, and how to vse bulles and other cattell to the yoake, and fell. With diuers approued remedies, to helpe most diseases among cattell: most necessarie for all, especially for husband men, hauing the gouernment of any such cattell (London: John Wolfe, 1587), 67 . For discussions on Mascall, see also: Cole, ‘Contagion’, 42; Spinage, *Cattle Plague*, 97. For popular veterinary texts, see: Louise Hill Curth , ‘“A Remedy for His Beast”: Popular Veterinary Texts in Early Modern England’, in Karl A. E. Enenkel and Mark S. Smith (eds.), Early Modern Zoology: The Construction of Animals in Science, Literature and the Visual Arts (Leiden: Brill, 2007), 369–87.

22

Theophilius Lobb , Letters Relating to the Plague and other Contagious Distempers (London: James Buckland, 1745), 369 .

23

Terence Ranger , ‘Plagues of beasts and men: prophetic responses to epidemic in eastern and southern Africa’, in Terence Ranger and Paul Slack (eds.), Epidemics and Ideas: Essays on the Historical Perception of Pestilence (Cambridge: Cambridge University Press, 1992), 244, 268.

24 See debates in the thirteenth and fourteenth centuries about whether animals diseases could infect humans: Samuel Cohn, ‘Idea of Contagion and the Black Death’ in James Kenneway and Rina Knoeff (eds.), *The Idea of Contagion* (forthcoming); Spinage, *Cattle Plague*, 36.

25 Charles Creighton, *A History of Epidemics in Britain from A.D. 664 to the Extinction of Plague* (Cambridge: Cambridge University Press, 1891), 218.

26
*Annals of the Kingdom of Ireland by the Four Masters from the Earliest Period to the Year 1616*, trans, John O’Donovan (Dublin: Hodges, Smith & Co., 1856), vol. 5, 1663; J. F. D. Shrewsbury , A History of Bubonic Plague in the British Isles (Cambridge: Cambridge University Press, 1970); Creighton, *History of Epidemics*, 565.

27
*A True bill of the whole number that hath died in the cittie of London, the citty of Westminster, the citty of Norwich, and diuers other places, since the time this last sicknes of the plague began in either of them, to this present month of October the sixt day, 1603 with a relation of many visitations by the plague, in sundry other forraine countries* (London: John Trundle, 1603). This is also repeated in: *The Red-Crosse: or, Englands Lord haue mercy vpon vs [A lament]able relation of many visitations by the plague in times past, as well in other countries as in the city of London, and the certaine causes thereof: with a true number of all those that dyed in the last great visitation, at the comming in of King Iames: and also the number of all those that haue dyed this present visitation; with two speciall medicines against the plague* (London: John Trundle, 1625).

28
*Flagellum dei, or, A collection of the several fires, plagues, and pestilential diseases that have hapned in London especially, and other parts of this nation from the Norman Conquest to this present, 1668* (London: C.VV, 1668), 2.

29 On this point for the clergy of the Low Countries, see: Sundberg, *Natural Disaster*, 212–50.

30 James Paterson, *A Warning to Great-Britain in a Sermon Preach’d at Several Churches in and about London, Upon the Spreading of the Plague in France, and Now Publish’d for the Benefit of Others* (London, s.a.), 38, 39–40, 58, 66.

31
*Gods three arrovves plague, famine, svvord, in three treatises. I. A plaister for the plague. II. Dearths death. III. The Churches conquest over the sword. By William Gouge Doctor in Divinity, and preacher of Gods Word in Black-Friers, London* (London: George Miller, 1631), 170–1.

32

Gideon Harvey , A discourse of the plague containing the nature, causes, signs, and presages of the pestilence in general, together with the state of the present contagion (London: Nathaniel Brooke, 1665), 1 .

33

Thomas Brooks , A heavenly cordial for all those servants of the Lord that have had the plague …, or, Thirteen divine maximes, or conclusions, in respect of the pestilence (London: John Hancock, 1666), 4 .

34

George Pye , A Discourse of the Plague; Wherein Dr. Mead’s Notions Are Consider’d and Refuted (London: J. Darby, 1721), 40 . Although Pye was an opponent of Mead who – as Margaret DeLacey notes – ‘implied…that Mead contributed to the spread of plague by causing unjustified terror, which led people to contract the disease’: DeLacey, *Germ of an Idea*, 159,

35

Richard Brookes , A history of the most remarkable pestilential distempers that have appeared in Europe for three hundred years last past; with what proved successful or hurtful in their cure (London, 1721), 13 . On Brookes and plague, see: DeLacy, *Germ of an Idea*, 158.

36

Lover of Mankind, A discourse concerning the plague, with some preservatives against it. By a lover of mankind. Dii taiem Terris avertite Pestem. Virgil (London: J. Roberts, 1721), 17 .

37
*An Act to oblige Ships Coming from Places Infected, More Effectually to Perform Their Quarentine* (1710). For the Baltic plague, see: Karl-Erik Frandsen , The Last Plague in the Baltic Region, 1709-1713 (Copenhagen: Museum Tusculanum Press, 2010). See: Charles Mullett , ‘A Century of English Quarantine, 1709-1825’, Bulletin of the History of Medicine, 23, 6 (1949), 527–4515397461 .

38 For recent studies of these measures, see: Demichel, *Rique et vigilance sanitaine*; Ermus, *Great Plague Scare*; Murphy, *Plague, Towns and Monarchy.* For British quarantine regulations of the early 1720s, see: Booker, *Maritime Quarantine*, chapter 4.

39

Richard Mead , A Short Discourse Concerning Pestilential Contagion and the Methods To be used to Prevent it (London: Sam Buckley, 1720). For a full discussion of the ideas which Mead puts forward in this treatise, see: DeLacy, *Germ of an Idea*, 153-57; Zuckerman, ‘Plague and Contagion’, 273–308; Mullett, *Bubonic Plague and England*, 275–82; idem, ‘English Plague Scare’, 484–516.

40 Blackmore was also an opponent of inoculation, for which Sir Richard Mead was an advocate and who had used condemned prisoners in Newgate to experiment with Chinese methods of smallpox inoculation: Rajesh Kochhar, ‘Smallpox in the modern scientific and colonial contexts 1721-1840’, *Journal of Biosciences*, 36, 5 (2011), 763.

41

Richard Blackmore , A Discourse Upon the Plague with a Preparatory Account of Malignant Fevers (London: John Clark, 1721), 27 . For a discussion of Blackmore’s ideas here, see: DeLacey, *Germ of an Idea*, 159–60.

42
*Distinct Notions of the Plague with the Rise and Fall of Pestilential Contagion. By the Explainer* (London: J. Peele, 1722), 100.

43

John Pringle , A rational enquiry into the nature of plague: drawn from historical remarks on those that have already happpen’d, That as the air only is capable of producing, or communicating it; the Method of Prevention now practis’d in France, is not only inhumane, but useless, and even pernicious (London: J. Peele, 1722), 12 . The remark ‘Polish Plagues’ is probably in reference to the fact that by the eighteenth century Poland had become Europe’s largest supplier of cattle: White, ‘A Model Disaster’, 106.

44

Peter Kennedy , A second discourse, by way of supplement to Dr. Kennedy’s first, on pestilence and contagion, Etc (London: T. Bickerton, 1721), 9 .

45
*A treatise of the plague contayning the causes, signes, symptomes, prognosticks, and cure thereof. Together with sundry other remarkable passages (for the prevention of, and preservation from the pestilence) never yet published by anie man. Collected out of the workes of the no lesse learned than experimented and renowned chirurgian Ambrose Parey* (London: R. Y. Young & R. C. Cotes, 1630), 11–12.

46 For the Parisian plague tract of 1348, see: Rosemary Horrox (ed.), The Black Death (Manchester: Manchester University Press, 1994), 158–62.

47

Samuel K. Cohn , Epidemics: Hate and Compassion from the Plague of Athens to AIDS (Oxford: Oxford University Press, 2018), 111–16.

48 ‘An abstract of a letter from Dr. Wincler chief Physitian of the Prince Palatine, dat. Dec. 22. 1682 to Dr. Fred. Slare Fellow of the Royal Society, containing an account of a Murren in Switzerland, and the method of its cure’, *Philosophical Transactions*, 13, 145 (10 March 1683), 93–4.

49 For Hodges’ ideas and how they interacted with those of Mead, see: Zuckerman, ‘Contagionism’, 280–1.

50
*Loimologia: or, An historical account of the plague in London in 1665: With Precautionary directions against the like contagion. By Nath. Hodges, M.D. and Fellow of the Royal Society of Physicians, who resides in the city all that time* (London: E. Bell & J. Osborn, 1720), 41.

51 Paterson, *Sermon Preach’d at Several Churches*, 38. Likewise, in his sermon during the 1665 plague in London William Gough wrote that cattle became infected by plague through eating ‘corrupt grasse’: *Gods three arrovves plague*, 170–1.

52

David Jennings , Behold the desolations in the earth! A sermon preached at Crosby-Square, Nov. 30. 1721. A time of solemn prayer, on occasion of the plague in France. By David Jennings. Published at the Desire of the Ministers who heard it (London: John Clark, 1721), 7 ; Randolph Ford , Divine threatenings, our most faithful monitors. Being the substance of two sermons: preach’d in the parish church of St. Mary la Bonne, Middlesex; on Friday the 16th, 1720. The Day Appointed for a General Fast and Humiliation; for Obtaining Pardon of Our Sins, and Averting God’s Judgements; and particulalry, for Beseeching the Almighty to Preserve Us from the Plague (London: Thomas Roycroft, 1721), 9 .

53 White, ‘A Model Disaster’, 113.

54
*Book of Husbandry by Master Fitzherbert*, 79; Spinage, *Cattle Plague*, 96.

55 Cited in Spinage, *Cattle Plague*, 37 (see also: 107–10 for a discussion of Lancisi’s methods).

56 Giovanni Maria Lancisi, *Dissertatio historica de bovilla peste, ex Campanaie finibus anno MDCCXIII Latio importata: deque praesidiis Maximum ad avertendam aëris labem, et annonae caritatem opportune adhibitis. Cui accedit consilium de equorum epidemia, quae Romae grassata est anno MDCCXII* (Rome: Salvioni, 1715). For discussions of Lancisi’s methods and ideas, see: Cole, ‘Contagion’, 42–3; Saul Jarcho , ‘A Papal Physician and the Sanitation of New York City’, Bulletin of the History of Medicine, 52, 3 (1978), 410–18376012 ; Wilkinson, ‘Rinderpest’.

57

Bernadino Ramazzini , De contagiosa epidemia, quae in Patavino agro, & tota fere Veneta ditione in boves irrepsit (Padua: G. B. Conzatti, 1712), 17 . See also: John M. McDonald , ‘Ramazzini’s dissertation on rinderpest’, Bulletin of the History of Medicine, 12, 3 (1942), 529–39.

58 Cited in: Wilkinson, ‘Rinderpest’, 132 (see this article more widely for a discussion of Ramazzini’s ideas). See also: Spinage, *Cattle Plague*, 108–9.

59 Bernardino Ramazzini, ‘An extract from the Acta Eruditorum for the Month of March, 1713’, *Philosophical Transactions*, 29, 338 (31 March 1714), 46–49. This was accompanied by published ‘A recipe: Or the ingredients of a medicine for the spreading mortal distemper among cows: lately sent over from Holland, where a like distemper raged amongst the black cattle’.

60 ‘An account of book, intituled, conghietture del Dottor Pietre Anton. Michelotti, Filosofo, e medico d’ arco, sopra la natura, cagione e rimedi dell’ infermità regnanti ne’ animali bovini di molte città, &c. Nell’ autunno del’ anno cadente, 1711. In Venezia, 1712’, *Philosophical Transactions*, 31, 365 (31 August 1720), 83–86.

61 For Fracastro’s ideas on contagion, see: Vivian Nutton , ‘The Reception of Fracastro’s Theory of Contagion: The Seed that Fell among Thorns?’, Osiris, 6 (1990), 196–234 11612689 10.1086/368701; Spinage, *Cattle Plague*, 59–60. For historic ideas about contagion, see: Vivian Nutton , “The Seeds of Disease: An Explanation of Contagion and Infection from the Greeks to the Renaissance’, Medical History, 27 (1983), 1–34 6339840 10.1017/s0025727300042241PMC1139262.

62 Athanasius Kircher, *Scrutinium Physico-Medicum Contagiosae Luis, que Pestis dicitur* (1658); Paula Findlen, ‘Microscopic Musings: Athanasius Kircher and the Roman Plague of 1656–57’, *Harvard Library Bulletin* (2022); Charles E. A. Winslow, *The Conquest of Epidemic Disease* (Princeton, 1980), 144–53.

63 Jean-Baptiste Bertrand, *Observation faites sur la peste qui règne à présent à Marseille et dans la Provence avec un avertissement* (Lyon: Laurens, 1721); DeLacey, *Germ of An Idea*, 151–2; Lisetta Lovett, ‘Dr Jean-Baptiste Bertrand’s Account of the Marseille Plague of 1720’, *Topics in the History of Medicine* 2 (2022), 17–37; Remi Barbieri et al, ‘The role of louse-transmitted diseases in historical plague pandemics’, *The Lancet Infectious Diseases*, 21, 2 (2021).

64 Hunniger, ‘Bugs, Worms and Dying Cattle’, 89–90.

65 Bradley’s ideas have been the object of much scholarly debate. See especially: DeLacy, *Germ of an Idea*, 160–2; Raymond Williamson , ‘The germ theory of disease; neglected precursors of Louis Pasteur: Richard Bradley, Benjamin Marten, Jean-Baptiste Goiffon’, Annals of Science, 11, (1955), 44–57 ; F. N. Egerton , ‘Richard Bradley’s illicit excursion into medical practice in 1714’, Medical History, 14, 1 (1970), 53–62 4904730 10.1017/s0025727300015131PMC1034014; Lise Wilkinson , Animals and Disease: An Introduction to the History of Comparative Medicine (Cambridge: Cambridge University Press, 1992), 47–8. For animate contagion, see: Catherine Wilson , The Invisible World: Early Modern Philosophy and the Invention of the Microscope (Princeton: Princeton University Press, 1995)10.1080/0003379960020043111616370, chapter 5.

66 Thomas Bates, ‘A brief account of the contagious disease which raged amongst the milch cowes near London, in the year 1714. And of the methods that were taken for suppressing it’, *Philosophical Transactions*, 30, 358 (31 December 1718), 884. On this passage, see also: Cole, ‘Contagion’, 44 (Lucinda Cole also notes that Bates had been based in Sicily when the disease first broke out: p. 43); Spinage, *Cattle Plague*, 111–12.

67
*A new discovery of the nature of the plague. And the true cause of its raging in European cities. With the remedy. Contrary to the opinion of Dr Meade, Dr Browne and others, who give for the First Causes of the Plague, in late Discourses, Air, Diet, and Disease* (London: Bickerton & Wilford, 1721), 12, 24. For Joseph Browne’s treatise, see: DeLacy, *Germ of an Idea*,

68 Lobb, *Letters Relating to the Plague*, 374–7, 383–9. For Lobb’s ideas contained in this passage, see: Cole, ‘Contagion’, 35–6; Margaret DeLacy , Contagionism Catches On: Medical Ideology in Britain, 1730-1800 (Cham: Palgrave Macmillian, 2017), 27 .

69 Francis Herring, *A modest defence of the caueat giuen to the wearers of impoisoned amulets, as preseruatiues from the plague wherein that point is somewhat more lergely reasoned and debated with an ancient physician, who hath mainteined them by publicke writing: as likewise that vnlearned and dangerous opinion, that the plague is not infectious, lately broched in London, is briefly glansed at, and refuted by way of preface* (London: Arnold Hatfield, 1604), 27–8. For the early modern classification of animals, see: Thomas, *Man and the Natural World*, 92–191.

70 Mead, *Pestilential Contagion*, 34.

71
*A new discovery of the nature of the plague*, 12, 24.

72 Mead, *Discourse*, 88.

73 Rose, *Succinct treastise of the plague*, 30, 31.

74
*Loimologia*, 41, 42, 58–9, 60–1, 142.

75

Philip Rose , A therico-practical, miscellaneous, and succinct treastise of the plague. Shewing its nature, signs, causes, prevention and cure (London: John Field, 1721), 11, 23.

76 For the provision of meat to the plague sick, see: Cohn, *Cultures of Plague*, 278–9; Jane L. Stevens Crawshaw , Plague Hospitals: Public Health for the City in Early Modern Venice (Farnham: Ashgate, 2012), 136–43; Neil Murphy , ‘Plague Hospitals, Poverty and the Provision of Medical Care in France, c.1450-c.1650’, Journal of Social History, 55, 4 (2022), 840–1.

77
*Medicina Flagellata: or the Doctor Scarify’d* (London, 1721), 195–6.

78 Pichatty de Croissante, *A brief journal of what passed in the city of Marseilles, while it was afflicted with the plague, in the year 1720. Extracted from the register of the council-chamber of the Town House, Counsellor and Orator of that City, and the King’s Attorney in Affairs relating to the good Government of it* (London: J. Roberts, 1721), 71.

79
*Lachrymæ Londinenses: or, Londons lamentations and teares for Gods heauie visitation of the plague of pestilence* (London: B. Alsop and T. Fawcet, 1626), 4.

80
*Loimologia*, 236. For Wolsey’s plague regulations, see: Paul Slack , The Impact of Plague in Tudor and Stuart England (Oxford: Oxford University Press, 1985), 201–2.

81
*A new discovery*, 47.

82 Cited in: Rawcliffe, ‘Animals’, 54–5.

83 Edwards, ‘Domesticated Animals in Renaissance Europe’, 87.

84 Rawcliffe, ‘Animals’; idem, *Urban Bodies*; Riguelle, ‘Purifying the Streets’.

85
*Medicina Flagellata*, 195–6.

86

Timothy P. Newfield , ‘Epizootics and the consumption of diseased meat in the Middle Ages’, in F. Ammannati (ed.), Religion and Religious Institutions in the European Economy 1000-1800 (Florence: Florence University Press, 2012), 619–39.

87
*Loimologia*, 42.

88

Mary Fissel , Imagining Vermin in Early Modern England’, History Workshop Journal, 47 (1999), 1–29 10.1093/hwj/1999.47.111623896.

89 Creighton, *History of Epidemics*, 166, 171.

90

Graham Twigg , The Black Death: A Biological Reappraisal (New York: Schocken Books, 1985).

91 Ranger, ‘Plagues of beasts and men’, 268.

92 François Vallat, *Les boeufs malades de la peste. La peste bovine en France et en Europe (XVIIIe-XIXe siècle)* (Rennes: Presses universitaires de Rennes, 2009), 136.

